# Periodicity of DNA in exons

**DOI:** 10.1186/1471-2199-5-12

**Published:** 2004-08-18

**Authors:** Stephen T Eskesen, Frank N Eskesen, Brian Kinghorn, Anatoly Ruvinsky

**Affiliations:** 1 Institute of Genetics and Bioinformatics, University of New England, Armidale, NSW, Australia; 2 Thomas J. Watson Research Center, Yorktown Heights, NY, USA

**Keywords:** Codon usage, DNA periodicity, simulated sequences, evolutionary algorithm

## Abstract

**Background:**

The periodic pattern of DNA in exons is a known phenomenon. It was suggested that one of the initial causes of periodicity could be the universal (**RNY)_n_**pattern (**R **= **A **or **G**, **Y **= **C **or **U**, **N **= any base) of ancient RNA. Two major questions were addressed in this paper. Firstly, the cause of DNA periodicity, which was investigated by comparisons between real and simulated coding sequences. Secondly, quantification of DNA periodicity was made using an evolutionary algorithm, which was not previously used for such purposes.

**Results:**

We have shown that simulated coding sequences, which were composed using codon usage frequencies only, demonstrate DNA periodicity very similar to the observed in real exons. It was also found that DNA periodicity disappears in the simulated sequences, when the frequencies of codons become equal.

Frequencies of the nucleotides (and the dinucleotide **AG) **at each location along phase 0 exons were calculated for *C. elegans*, *D. melanogaster *and *H. sapiens*. Two models were used to fit these data, with the key objective of describing periodicity. Both of the models showed that the best-fit curves closely matched the actual data points. The first dynamic period determination model consistently generated a value, which was very close to the period equal to 3 nucleotides. The second fixed period model, as expected, kept the period exactly equal to 3 and did not detract from its goodness of fit.

**Conclusions:**

Conclusion can be drawn that DNA periodicity in exons is determined by codon usage frequencies. It is essential to differentiate between DNA periodicity itself, and the length of the period equal to 3. Periodicity itself is a result of certain combinations of codons with different frequencies typical for a species. The length of period equal to 3, instead, is caused by the triplet nature of genetic code. The models and evolutionary algorithm used for characterising DNA periodicity are proven to be an effective tool for describing the periodicity pattern in a species, when a number of exons in the same phase are analysed.

## Background

Periodicity of DNA in exons, with the period being equal to 3 nucleotides, has been well known for some time [1-6]. This periodicity reflects correlations between nucleotide positions along coding sequences [7], which is caused by the asymmetry in base composition at the three coding positions [8]. This periodicity has also been suggested as a reading-frame monitoring device during translation, due to interrupted periodic patterns matching with frame shifts downstream where the periodic pattern returns [9].

The triplet code has undergone evolution itself, from the earliest form of the triplet code to what exists today. The universal DNA periodicity observed in exons suggests a (**RNY)_n_**pattern (**R **= **A **or **G**, **Y **= **C **or **U**, **N **= any base), which probably was inherited from the earliest mRNA sequences [10,11].

In this study comparisons between real and simulated coding sequences were used in attempt to better understand the cause of the DNA periodicity. The only data used by the simulation program were codon usage frequencies from real species. Thus the simulated coding sequences had frequencies of codons very similar to real species. The major difference, however, was a random position of codons in simulated sequences.

The periodicity of exons, as well as other coding statistics can be an additional tool for exon prediction programs [7]. The distance between two types of nucleotides is counted, and a period is determined by the distance between the similar frequencies. For example, if there is a nucleotide **A **at one point in a sequence, and other **A**'s are more common when there are 2, 5, 8 and so on nucleotides between them, a period of three can be determined [7]. Additional methods of finding periodicity include Fourier analysis [12], the length shuffle Fourier transform algorithm [13], autocorrelation functions [14] and distance analysis [15]. We applied two models of an evolutionary algorithm [16,17] to quantify DNA periodicity. Thus the second objective of this study was investigation of a new method for quantification of DNA periodicity.

## Results

### Periodicity of DNA in exons

As mentioned in the Background, periodic 3-nucleotide pattern has been known for eukaryotic exons for some time. We studied a question whether DNA periodicity similar to that observed in exons can be simulated in computer experiments utilising codon usage frequencies (CUF) of real species as the only source of information. The computer program GENERATE, which was used in these experiments, composed artificial coding sequences using CUF of several species as the only source of information. Thus despite random choice the frequencies of codons in simulated sequences were very similar to the real CUF.

**Figure 1:**
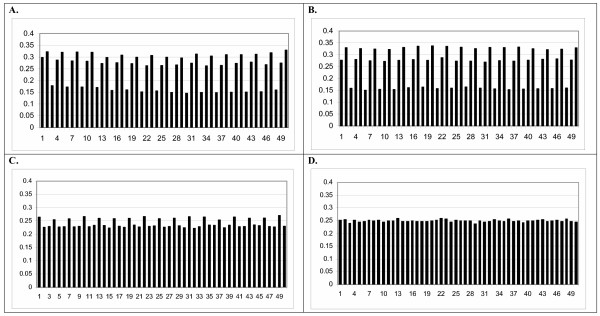
**A. Adenine **periodicity in *Drosophila melanogaster *exons (N_exons _= 32,760); **B. Adenine **periodicity in simulated coding sequences (N_genes _= 13,065) created by GENERATE using *D. melanogaster *CUF; **C. Adenine **periodicity in simulated coding sequences (N_genes _= 13,065) created by GENERATE using equal frequencies of all non-stop codons and *D. melanogaster *frequencies of stop codons and **D. Adenine **periodicity in simulated coding sequences (N_genes _= 13,065) created by GENERATE using equal frequencies of all codons including frequencies of stop codons. The length of coding sequences was determined by the program.

As an example of these experiments, Figure 1A shows distribution of **Adenine **nucleotides in real *Drosophila melanogaster *exons (phase 0) and in simulated (Figure 1B) coding sequences (phase 0) created by GENERATE using *D. melanogaster *CUF. To avoid any significant influences of splicing signals, *D. melanogaster *exons aligned at the 5' end start from the 10^th ^nucleotide (4^th ^codon). In the simulated coding sequences periodicity is also highly pronounced and the periodicity patterns observed in *D. melanogaster *exons and simulated sequences are nearly identical (Figure 1A &1B). Other studied species *C. elegans *and *H. sapiens *despite significant differences in **AT **and **GC **content also show high similarity in the periodicity pattern between exons and simulated sequences (data are not shown). Periodicity of other nucleotides was also observed and it shown high similarity in both DNA of real exons and simulated sequences (data are not shown). The obvious conclusion following from this study is that CUF, which was the only source of information for the simulated coding sequences, is the crucial factor determining periodicity.

DNA periodicity in simulated coding sequences was dramatically reduced in the experiments where the frequencies of all non-stop codons were made equal (Figure 1C). This observation strongly supports the conclusion that codon usage frequencies determine DNA periodicity in exons. A very light periodicity of **Adenine **and **Thymine **(data are not shown) was caused by the fact that 3 stop codons and the corresponding combinations of nucleotides were present in the simulated coding sequences in different and much lesser frequencies than other codons. **Cytosine**, which is not a component of any stop-codon, does not show periodic pattern at all because frequencies of **Cytosine **containing codons were equal to frequencies of all other non stop codons. Finally, when frequencies of all codons, including stop codons, were made equal, no periodicity was observed in the simulated sequences (Figure 1D).

Thus the computer simulations lead to a firm conclusion that 3 nucleotide periodicity observed in DNA of exons is determined by codon usage frequencies. The triplet nature of genetic code is rather responsible for the length of the period but not periodicity itself, as some people might think.

### Quantification of DNA periodicity using an evolutionary algorithm

Data sets on frequency of the nucleotides and all 16 dinucleotides at each location were constructed for *Caenorhabditis elegans*, *Drosophila melanogaster *and *Homo sapiens *phase 0 exons. The frequencies of **Adenine **and dinucleotide pair **AG **are shown in this paper as an example. Two models were used to fit to these data, with the key objective of describing periodicity in the data.

**Figure 2:**
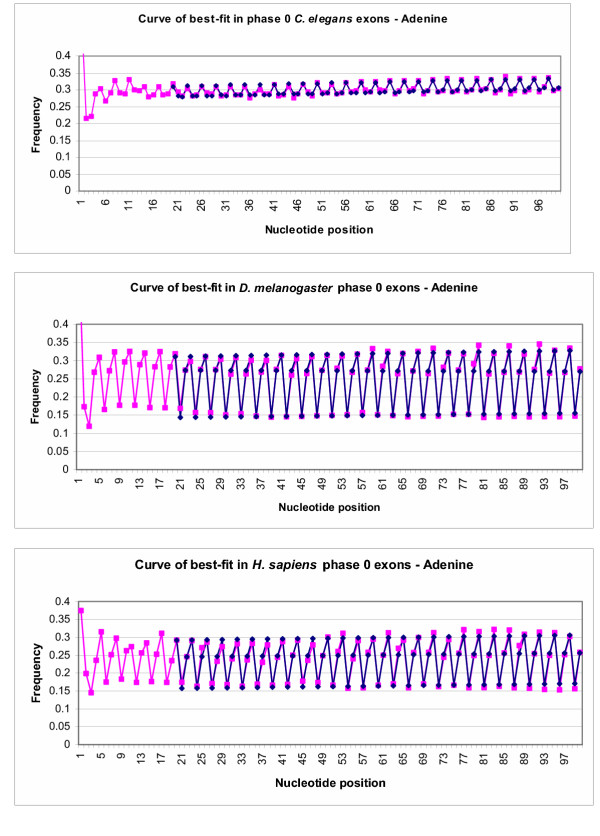
Curves of best-fit for **Adenine **in phase 0 exons compared to actual frequencies. Pink points represent frequencies of nucleotide A in phase 0 *C. elegans*, *D. melanogaster *and *H. sapiens *exons aligned at the 5' end. Blue points represent the best-fit curve for the data points in an ideal situation, from position 20–100. The scales of the graphs were altered to provide better contrast between the data points.

**Figure 3:**
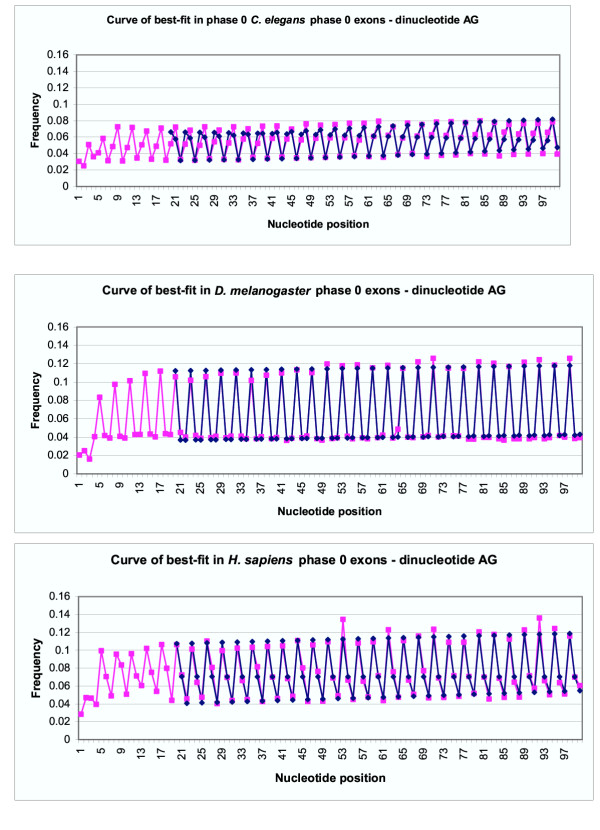
Curves of best-fit for dinucleotide **AG **in phase 0 exons compared to actual frequencies. Pink points represent frequencies of AG in phase 0 *C. elegans D. melanogaster *and *H. sapiens *exons aligned at the 5' end. Blue points represent the best-fit curve for the data points in an ideal situation, from position 20–100. The scales of the graphs were altered to provide better contrast between the data points.

**Figure 4:**
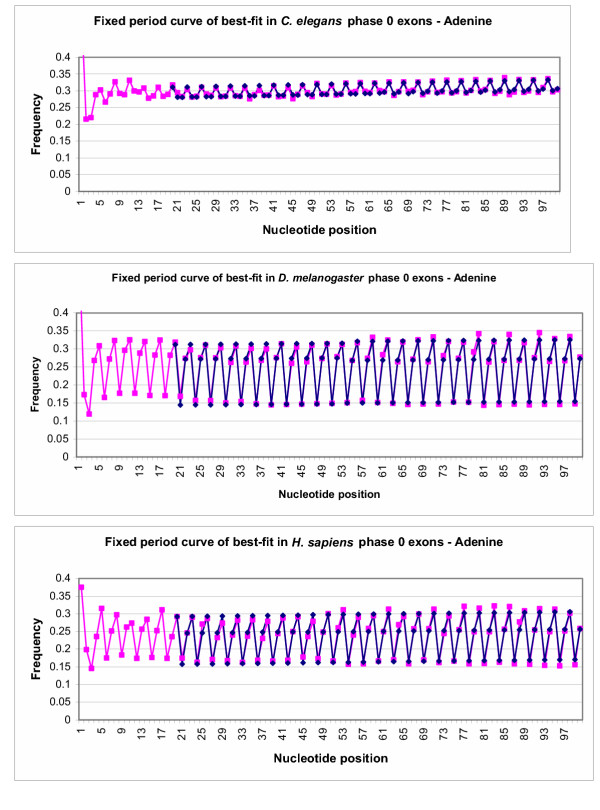
Fixed period best-fit curves for **Adenine **in phase 0 exons compared to actual frequencies. Pink points represent frequencies of nucleotide A in phase 0 exons aligned at the 5' end. Blue points represent the best-fit curve for the data points in an ideal situation, from position 20–100. The scale of the graph was altered to provide better contrast between the data points.

**Figure 5:**
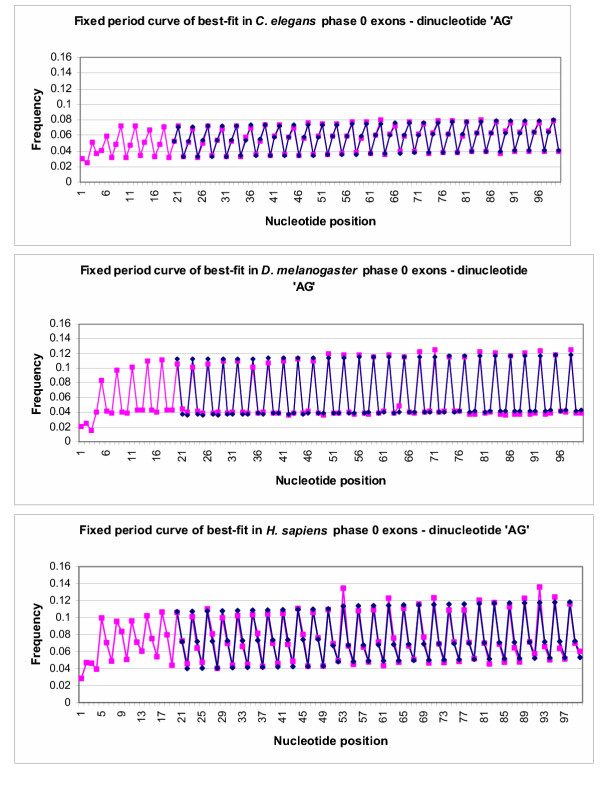
Fixed period best-fit curves for dinucleotide **AG **in phase 0 exons compared to actual frequencies. Pink points represent frequencies of dinucleotide **AG **in phase 0 exons aligned at the 5' end. Blue points represent the best-fit curve for the data points in an ideal situation, from position 20–100. The scale of the graph was altered to provide better contrast between the data points.



Inspection of Figures 2 to 5 shows that periodicity is quite apparent, and that the frequencies between peak and trough frequencies are generally consistent, but sometimes trending in value. Two models were used to accommodate this shifting pattern. The first model is:

- where  is predicted frequency at nucleotide position *i*, and *b*_1 _to *b*_5 _are parameters to be estimated from data. Because of irregularities in frequencies close to the 5' end of exons, nucleotide position 20 was take as position *i *= 1.

The component *b*_1 _+ *b*_2_*i *fits an overall linear trend in frequencies, independently from finer-scale periodicity. For some data sets it could be useful to include a quadratic term for *i*.

Parameter *b*_3 _gives the amplitude of periodic waves. As *2π *radians describes a full cycle, periodicity is given by parameter *b*_5_. However, if *b*_5 _differs from exactly 3, this generates a shift in phase that is linear with nucleotide position. This shift combines with static phase shift parameter *b*_4 _to fit the relative frequencies in adjacent groups of three locations. This is not an ideal model, in that parameter *b*_5 _does not cleanly describe periodicity, but it proves to work well in practice.

The second model is:



- where the value of Offset depends on nucleotide position I as follows:

if i < b4 Then Offset = b6

else if i < b4 + b5 Then Offset = b7

else Offset = b8

Thus, in this case, three regions are defined by parameters *b*_4 _and *b*_5_, and Offset is defined within region by parameters *b*_6 _and *b*_7_.

Model 2 fixes periodicity at 3 nucleotides, but allows for different patterns of relative frequency in regions chosen by the data.

The analysis task for both models is to find values of the *b *parameters that give a close fit between the real frequencies *Y *and the predicted frequencies . The criterion used for this was the sum of squared errors across nucleotide position:



Best-fitting *b *parameters were found using a form of evolutionary algorithm (Differential evolution, [17]) with modifications to improve robustness following [18].

To test if the above method could identify a quantifiable period in exons, several tests were performed. All phase 0 exons were extracted from the EID database, described in Material and Methods. This procedure dramatically enhanced the visible periodicity compared to exons of all three phases (Figure 1A.). Once the phase 0 exons were separated, they were aligned at the 5' end and data for all four single nucleotide frequencies were analysed using both methods. In addition to all single nucleotide frequencies, the dinucleotide frequency of **AG **was run through the analysis for both dynamic period determination best-fit curve, and the static period of 3 best-fit curve. This was done for the three studied species, *C. elegans*, *D. melanogaster *and *H. sapiens *for phase 0 exons. Several other dinucleotide pairs also shown clear periodicity patterns in exons (data are not shown). **AG **was used in this paper as an example. Introns were only run through the analysis for dynamic period determination and did not show clear and stable periodicity.

### Phase 0 exons – dynamic period determination model (Model 1)

Curves of best-fit were created for the four separate nucleotides and the dinucleotide **AG **for *C. elegans*, *D. melanogaster *and *H. sapiens *phase 0 exons. These curves were created using model 1, in an attempt to find a periodicity within the given data. The first few positions of exons are frequently under different selection pressures, which do not always conform to the same pressures as the remainder of the exon. It was for this reason that the algorithm was run starting from position 20 to position 100 in exons.

Table 1, shows DNA periodicity in exons of *C. elegans*, *D. melanogaster *and *H. sapiens *as they were determined by the analysis. The amplitude of the periodicity was measured as the variation from the center-point of the sine curve. As mentioned earlier, the criterion value of goodness of fit is the sum of squared deviations between observed frequencies and frequencies predicted by the model with the prevailing parameters. This means that as the analysis runs through its generation cycles, it finds better fitting curves as it goes along and replaces the previous curve of best-fit. The criterion value is a reflection of this process, in that as it gets closer to zero, the closer the curve of best-fit represents actual data. The data show that in all cases, the determined period is very close to 3 in exons for all three species for all nucleotides and the dinucleotide pair **AG**. The range of periods goes from a low of 2.990391 in *C. elegans *nucleotide **C**, a difference from 3 is ~0.0096, to a maximum of 3.019349 in *C. elegans *dinucleotide pair **AG**, a difference from three of ~0.0193. The criterion values of goodness of fit are also very low for exons, with the largest among them at 0.0093573 in *H. sapiens *nucleotide **C**.

**Table 1:** Periods of best-fit curves in phase 0 aligned exons. Periods and criterion values of phase 0 *C. elegans D. melanogaster *and *H. sapiens *exons aligned at the 5' end. The four single nucleotide as well as a single dinucleotide pair, **AG **were studied.

**Species**	**Nucleotides**	**Best-Fit Period**	**Amplitude**	**Criterion Value**
*C. elegans*	**A**	2.995367	± 0.02007	0.0019226
	**C**	2.990391	± 0.01583	0.0019602
	**G**	3.005597	± 0.08199	0.0047119
	**T**	3.014848	± 0.06247	0.0049716
	**AG**	3.019349	± 0.02077	0.0031441
*D. melanogaster*	**A**	3.002192	± 0.10128	0.0055213
	**C**	3.000776	± 0.07056	0.0043703
	**G**	2.998296	± 0.09142	0.0036970
	**T**	2.999109	± 0.06115	0.0049037
	**AG**	2.999744	± 0.05038	0.0012143
*H. sapiens*	**A**	3.000390	± 0.07898	0.0087358
	**C**	2.997380	± 0.05627	0.0093573
	**G**	2.998682	± 0.08669	0.0014469
	**T**	2.998730	± 0.06627	0.0065035
	**AG**	2.995860	± 0.03872	0.0025106

A comparison between the best-fit curves for nucleotide **A **and actual frequencies of nucleotide **A **in exons of the three studied species can be seen in Figure 2. Figure 3 shows a similar comparison for the dinucleotide pair **AG**. Best-fit curves for nucleotides **C**, **G **and **T **are not shown. The blue points on the graph represent data points predicted by fitting model 1 to actual data, represented by pink points. As can be seen in the graphs, the blue best-fit curves in both Figure 2 and Figure 3 both closely follow the pink line for actual data, which confirms that the best-fit line quite accurately portrays actual data.

### Phase 0 exons – static period 3 model (Model 2)

The data were then fitted to model 2, keeping the period fixed at 3. With this algorithm, the ideal best-fit curve would remain nearly in the same pattern. Again phase 0 exons were used as sample data. Figure 4 shows the best-fit curve for nucleotide **Adenine **in *C. elegans D. melanogaster *and *H. sapiens *phase 0 exons with the period fixed at three. Figure 5 shows the best-fit curve for the dinucleotide pair **AG **in 0 exons in the same species. In both sets of graphs, pink points represent actual frequencies of nucleotides and the dinucleotide pair, **AG**, while the blue points represent the optimized curve of best-fit for these frequencies. It is clear from the graph that keeping the period fixed at exactly three in exons does not detract from the accuracy of the curve of best-fit. The curve of best-fit is remarkably similar to the actual data points.

## Discussion

The fact of DNA periodicity in exons, as well as the lack of periodicity in introns, is known for some time [1-6]. "Such a periodic pattern reflects correlations between nucleotide positions along coding sequences (that is, the probability of finding a nucleotide at a given position in a coding sequences is not independent of the nucleotide occurring at some other even distant position). The correlations arise, in turn, because of the asymmetry in base composition at the three codon positions in coding sequences" [7,8]. The simulation experiments described in the paper support such conclusion and provide a clear proof that frequency of codon usage is the key cause for DNA periodicity in exons (Figure 1). We have shown that simulations, which utilized only codon usage frequencies data, produced an exceptionally good match to periodicity observed in real exons. As soon as frequencies of all codons are set as equal, DNA periodicity in exons entirely disappears. It is reasonable to think that the asymmetry in base composition, studied by Guigó [7], might be caused by the codon usage frequency.

The results presented in this paper also demonstrate effectiveness of the evolutionary algorithm and the both models used to identify a periodicity pattern in exons. Although periods, which are seen in Table 1, are not precisely equal to 3, they are very close. This minor discrepancy is a result of the analysis compensating for slight changes in the pattern of frequencies over nucleotide position. When the period is fixed at exactly 3, and the program allows for change-over points where the curve of best-fit is adjusted to better suit the data, the curves of best-fit still closely match the actual data points (see Figures 4 and 5), revealing that the period of 3 is not simply coincidental when it is allowed to be determined by the program. As it can be seen in Figures 2,3,4,5 the amplitude of variation is much more narrow for *C. elegans *than for the two other species under consideration.

Introns do not show any specific period that can be determined by the analysis (results are not shown). Although the analysis does produce a period for each data set given, these periods are not consistent with each other, and the predictions do not fit the data well. As introns are not composed from codons, this is an additional indication supporting the conclusion that CUF determine periodicity pattern in exons.

Since only exons show a strong periodicity of three, this type of analysis can be in principle used as an additional component of exon finding tools. Such possibility was already considered [7]. Unfortunately, the methods discussed here being very effective in quantifying DNA periodicity in a set of many sequences, are not sensitive enough for a single sequence. Further modifications of the approach are necessary before it can be used in exon prediction programs.

## Conclusions

Conclusion can be drawn that DNA periodicity in exons is determined by codon usage frequencies. It is essential to differentiate between DNA periodicity itself, and the length of the period equal to 3. Periodicity itself is a result of certain combinations of codons with different frequencies typical for a species. The length of period equal to 3, instead, is caused by the triplet nature of genetic code. The models and evolutionary algorithm used for characterising DNA periodicity are proven to be an effective tool for describing the periodicity pattern in a species, when a number of exons in the same phase are analysed.

## Methods

### Exon-Intron Database

Information relevant to *C. elegans*, *D. melanogaster *and *H. sapiens*, was extracted from the exon-intron database (EID), which was compiled in the W. Gilbert laboratory, Department of Molecular and Cellular Biology, Harvard University [18]. The database contains protein-coding intron-containing genes. From the version of the database that we used, the following data were extracted: *C. elegans *14,836 genes and 98581 exons; *D. melanogaster *13,361 genes and 58,801 exons, *H. sapiens *7150 genes and 47908 exons.

### exScan

This program calculates frequencies of nucleotides or any combination of nucleotides in a database. exScan align all exons in the database at either the 5' or 3' end. The program then searches the exons for given sequences and give a summary of the sequences found. exScan was used in order to obtain the frequencies for nucleotides and dinucleotide pairs in each position of exons aligned at the 5' end. A summary of the program's operation follows:

- Command line for exScan selects the database to be used for searching.

- The string(s) to be searched are also entered into the command line.

- ExScan then aligns the exons by the 5' end and searches each exon for all matches to the search strings.

- The output of the program will provide the number of matches for each search string entered at every position along the aligned exons.

- These numbers are then converted into frequencies.

ExScan is written in the C++ programming language; its full description and the program itself are available upon request

#### GENERATE

The program simulates a required number of coding sequences, using as an input file CUF for a particular species and a generator of random numbers. Inclusion of stop-codon in a coding sequence terminates the gene. There are options, which allow establishing a minimal and a maximal length of genes as well a shape of gene length distribution. GENERATE was used in this study to show how CUF alone could create periodicity, even in randomly created sequences, which do not code for any real protein. The procedure when running GENERATE follows:

- GENERATE accepts as input a file containing usage frequencies of all 64 codons. These codon usage frequencies for different species were taken from the database located at . Thus, despite random choice the frequencies of codons in simulated sequences were very similar to the real CUF.

- These frequencies are then used to construct a requested number of artificial genes, with the codons chosen randomly based on their frequencies.

- Artificial genes all start with **ATG**, and will terminate once a stop codon is randomly chosen.

- The artificial genes can then be used as a separate database for analysis with exScan as above.

GENERATE was written in the C++ programming language. Description of the program and program itself are available upon request.

### Differential Evolution

The specific method used for fitting the two periodicity models was Differential Evolution (DE). As DE is a widely applicable method of general utility for optimization, the reader is directed to Storn and Price [13] for detailed description and example computer code. The concept is outlined here:

- A population of candidate solutions is established. Each population member is constituted by a randomly sampled set of *b *parameters and is characterized by its *fitness *(its value on the prevailing objective function, the sum of squared errors across nucleotide position).

- For each population member, a challenger is constructed. If this challenger has superior fitness, it will replace the population member in the next *generation*. A challenger is constructed as follows:

- Three other population members are chosen at random. We can label these as *a*, *b *and *c*. Each parameter is then addressed in turn. With probability *CR *(*CR *= 0.4 was adopted) the parameter is simply taken from the population member that the challenger is challenging. Otherwise, a new parameter value is constructed as the value for member *a *plus *F *times the *difference *of the values for *b *and *c*. For this application, *F *= 0.4, except *F *= 1 every fourth generation and F = 2 every seventh generation, to help avoid local optima. In addition, mutation independent from differences between other solutions was invoked periodically, also to help avoid local optima.

- Successful challengers replace their respective population members, and, together with surviving members constitute a new generation with higher mean fitness. The process continues over sufficient generations to achieve convergence close to an optimal solution, with the most fit solution being chosen.

## Competing interests

None declared.

## Authors' contributions

SE conducted the data analysis and was involved in drafting the manuscript. FE designed and wrote the code for the C++ computer programs. BK assisted with the manuscript and wrote the code for modeling and fitting periodicity. AR drafted the manuscript, performed statistical analysis, conceived the study and participated in its design and coordination. All authors read and approved the final manuscript.
